# Gastrointestinal Autonomic Neuropathy Exacerbates Gut Microbiota Dysbiosis in Adult Patients With Type 2 Diabetes Mellitus

**DOI:** 10.3389/fcimb.2021.804733

**Published:** 2022-02-08

**Authors:** Yuhui Du, Qiongli Neng, Yu Li, Yongbo Kang, Liqiong Guo, Xinwei Huang, Minghui Chen, Fan Yang, Jingan Hong, Shuai Zhou, Jianhua Zhao, Fubing Yu, Heng Su, Xiangyang Kong

**Affiliations:** ^1^ Medical Faculty, Kunming University of Science and Technology, Kunming, China; ^2^ Endocrinology Branch, The First People’s Hospital of Yunnan Province, Kunming, China; ^3^ School of Medicine, Southern University of Science and Technology, Shenzhen, China; ^4^ School of Basic Medical Sciences, Shanxi Medical University, Taiyuan, China; ^5^ Nutrition Department, The First People’s Hospital of Yunnan Province, Kunming, China; ^6^ Neurosurgery Department, The First People’s Hospital of Yunnan Province, Kunming, China; ^7^ Digestive System Department, Affiliated Hospital of Yunnan University, Kunming, China

**Keywords:** gut microbiota, type 2 diabetes mellitus, gastrointestinal autonomic neuropathy, gastrointestinal symptoms, diagnosis

## Abstract

**Objective:**

The diabetic autonomic neuropathy is one of the most common complications in type 2 diabetes mellitus (T2DM), especially gastrointestinal autonomic neuropathy (GAN), which occurs in up to 75% of patients. The study aimed to investigate the gut microbiota composition, structure, and function in T2DM patients with GAN (T2DM_GAN) and set up a link between gut microbiota and clinical characteristics of patients.

**Methods:**

DNA was extracted from fecal samples of three groups using the kit method: healthy volunteers (n = 19), the patients with T2DM (n = 76), and T2DM_GAN (n = 27). Sequencing of 16S ribosomal DNA was performed using the MiSeq platform.

**Results:**

According to the clinical data, higher age, lower triglyceride, and lower body mass index were the main features of patients with T2DM_GAN. The gut microbiota analysis showed that Bacteroidetes, Firmicutes, and Proteobacteria constituted the three dominant phyla in healthy individuals. In addition, the gut microbiota structure and function of T2DM_GAN patients were clearly different from that of T2DM patients. T2DM patients were characterized by Fusobacteria, Fusobacteriia, Fusobacteriales, Fusobacteriaceae, *Fusobacterium*, *Lachnoclostridium*, and *Fusobacterium_mortiferum*. Those gut microbiota may be involved in carotenoid and flavonoid biosyntheses. Relatively, the Gammaproteobacteria, Enterobacteriales, Enterobacteriaceae, *Escherichia-Shigella*, *Megasphaera*, *Escherichia_coli*, and *Megasphaera_elsdenii* were characteristic in the T2DM_GAN patients. Those may be involved in bacterial invasion of epithelial cells and pathogenic *Escherichia coli* infection.

**Conclusions:**

GAN exacerbated gut microbiota dysbiosis in adult patients with T2DM. The findings indicated that phyla Fusobacteria and class Gammaproteobacteria were closely related to the occurrence of T2DM. Especially the latter may promote T2DM_GAN.

## Introduction

With the increasing incidence of diabetes, diabetes complications have also increasingly become one of the most critical health problems in the world today (Cho et al., 2018; Meldgaard et al., 2018). Once diabetes occurs, it can cause various complications and a negative impact on the whole organism (Leustean et al., 2018). In general, the common complications of diabetes include macrovascular, peripheral vascular, and microvascular diseases (Leustean et al., 2018). One of the most common microvascular complications is neuropathy, including autonomic and peripheral neuropathy, which causes harmful changes in neurons’ structure and function (Meldgaard et al., 2018). Especially in neuropathy associated with alterations in the enteric nervous system has the highest symptom burden, since it affects a large proportion of patients with diabetes. A typical example is gastrointestinal autonomic neuropathy (GAN), which occurred in 75% of diabetic patients (Bytzer et al., 2001; Asgharnezhad et al., 2019). Those patients may experience a range of adverse gastrointestinal symptoms, such as postprandial fullness, nausea, vomiting, bloating, abdominal pain, diarrhea, and constipation (Bytzer et al., 2001; Asgharnezhad et al., 2019). A large cohort study has found that moderate to severe symptoms were associated with more inadequate glycemic control (Gatopoulou et al., 2012). Although heavily documented, GAN remains underrecognized and poorly treated in type 2 diabetes mellitus (T2DM) patients.

The pathophysiology of T2DM with GAN (T2DM_GAN) is complex and multifactorial. Although there is some clinical guidance on T2DM_GAN, there are still many knowledge gaps about the underlying specific pathogenesis and mechanism. There are multiple levels of regulation in the gastrointestinal tract: the central, autonomic, enteric nervous systems, the interstitial cells of Cajal (mainly “pacemaker cells”), and gut microbiota (Kempler et al., 2016). It is well known that there are significant changes in the composition of the microbiota in T2DM patients, and the altered microbiota likely contributes to T2DM pathogenesis (Gurung et al., 2020). Simultaneously, some gastrointestinal movement disorders, for example, diarrhea, have been attributed to change in the microbiota (Yarandi and Srinivasan, 2014). Therefore, the effect of gut microbiota on gastrointestinal motility has also attracted more and more attention. To date, there is little published information on gut microbiota of T2DM_GAN patients, and the exact role of altered microbiota in diabetes-induced gastrointestinal dysmotility has still not been investigated.

To investigate gut microbiota of T2DM_GAN, we collected stool samples from the healthy population, patients with T2DM, and T2DM_GAN and measured their gut microbiota by next-generation sequencing. Through comparing qualitative and quantitative changes in the intestinal flora of the three groups, we expected to screen flora and related functions contributed to the onset of T2DM_GAN.

## Subjects and Methods

### Standard Protocol Approvals and Patient Consents

The ethics review committee of The First People’s Hospital of Yunnan Province granted this study’s approval. Informed consent was obtained from all the subjects.

### Study Population

Participants in the trial were recruited from the Endocrinology department of the First People’s Hospital of Yunnan province in Kunming, China. All the eligible patients had an established diagnosis of T2DM, and the subjects included men and women. Inclusion criteria were the following: (1) age 20–75 years, (2) provision of written informed consent, and (3) meeting the 1999 World Health Organization (WHO) diagnostic criteria for T2DM. Exclusion criteria were (1) systemic antibiotics within 6 weeks before inclusion; (2) use of probiotics, prebiotics, and synbiotic within 3 months before inclusion; (3) daily alcohol consumption >30 g; (4) significant immunodeficiency; (5) serious kidney disease serious; (6) liver disease excluding fatty liver; (7) known cardiac valvular disease; and (8) breastfeeding or pregnancy. In addition, considering the influence of daily diet on gut microbiota, we make sure participants had relatively similar diet structure by questionnaires. Namely, nutritional habits will be assessed using a standardized 14-day recall questionnaire, which will be discussed with a dietician.

The demographic data for the patients with T2DM and T2DM_GAN are summarized in **Table 1**. The subjects were divided into three groups: T2DM group (n = 73), T2DM_GAN group (n = 27), and normal group (n = 19). Healthy controls included age- and sex-matched cohort with no known disease symptoms.

**Table 1:** Characteristic of the patients with T2DM and T2DM-GAN in the study.

Clinical parameters	T2DM	T2DM_GAN	*p-*value
Sex (male/female, n)	61/12	13/14	–
BMI (kg/m^2^)	27.13 ± 3.53	24.23 ± 3.18^***^	3.01×10^−4^
Age (year)	38.39 ± 4.01	53.44 ± 10.66^***^	7.56×10^−8^
ALT (U/L)	30.09 ± 19.28	25.52 ± 13.55	0.26
AST (U/L)	20.62 ± 9.01	24.67 ± 20.24	0.324
TBIL (μmol/L)	13.42 ± 4.57	11.03 ± 5.36^*^	0.029
DBIL (μmol/L)	4.20 ± 1.72	3.84 ± 1.95	0.378
UBIL (μmol/L)	9.26 ± 3.36	7.19 ± 3.83^**^	0.009
T_CHOL (mmol/L)	4.81 ± 1.39	4.36 ± 1.07	0.137
Triglyceride (mmol/L)	4.10 ± 4.04	1.91 ± 1.23^***^	5.21×10^−5^
HDL-C (mmol/L)	0.99 ± 0.69	0.99 ± 0.26	0.985
LDL-C (mmol/L)	2.61 ± 0.94	2.56 ± 0.81	0.779
FBG (mmol/L)	9.30 ± 3.77	8.20 ± 3.28	0.184
Fructosamine (mmol/L)	405.21 ± 107.28	371.96 ± 92.74	0.159
HbA1c (%)	10.13 ± 2.71	9.36 ± 1.92	0.178
BUN (mmol/L)	4.46 ± 1.11	5.35 ± 1.72^**^	0.003
Creatinine (μmol/L)	69.86 ± 14.07	65.93 ± 20.33	0.276
Uric acid (μmol/L)	366.72 ± 86.39	381.19 ± 105.91	0.486
WBC (10^9^/L)	6.76 ± 1.41	6.12 ± 1.42^*^	0.049
Neutrophils (10^9^/L)	3.68 ± 0.97	3.32 ± 1.26	0.143
Lymphocytes (10^9^/L)	2.45 ± 0.70	2.22 ± 0.54	0.120
Monocytes (10^9^/L)	0.42 ± 0.13	0.39 ± 0.10	0.328
Eosinophil (10^9^/L)	0.17 ± 0.14	0.18 ± 0.19	0.940
Basophil (10^9^/L)	0.03 ± 0.02	0.03 ± 0.02	0.635
RBC (10^12^/L)	5.13 ± 0.44	4.54 ± 0.46^***^	9.45×10^-8^
Hemoglobin (g/L)	156.73 ± 16.64	139.15 ± 15.13^***^	5.66×10^-6^
Hematocrit	0.46 ± 0.04	0.41 ± 0.04^***^	2.37×10^-7^
Platelets (10^9^/L)	204.79 ± 54.23	205.00 ± 68.54	0.988
Plateletcrit	0.24 ± 0.05	0.23 ± 0.07	0.418

BMI, body mass index; ALT, alanine aminotransferase; AST, aspartate aminotransferase; TBIL, total bilirubin; DBIL, direct bilirubin; UBIL, unconjugated bilirubin; T_CHOL, total cholesterol; HDL_C, high-density lipoprotein cholesterol; LDL_C, low-density lipoprotein cholesterol; FBG, fasting blood glucose; BUN, blood urea nitrogen; WBC, white blood cells; RBC, red blood cells

Data are presented as mean ± SD. P value is calculated using a two-tailed Student’s t test.

***p ≤ 0.001, **p ≤ 0.01, *p ≤ 0.05.

### Sample Collection and Processing

Height and body weight were measured, and body mass index (BMI) was calculated at the beginning of the study. Blood samples were also collected at recruitment and then detected serum biochemical and blood analysis. The fecal samples were collected and transported to the laboratory using an icebox, stored at −80°C, and extracted DNA for microbiota analysis. Fecal samples were collected from healthy volunteers, who had no abnormality as determined by medical examination.

### 16S Amplicon Preparation

Microbial DNA was extracted from each stool sample using the QIAamp DNA Stool Mini Kit (Qiagen, USA) as per the manufacturer’s instructions. DNA quality was assessed by agarose gel electrophoresis and NanoDrop™ One. The 16S rRNA gene V4 region was amplified using forward primer F-5′CCTACGGGRSGCAGCAG3′ and reverse primer R-5′GGACTACVVGGGTATCTAATC3′. Amplicon library was created for each sample and performed according to the Illumina MiSeq 16S metagenomic sequencing library preparation protocol. Paired-end sequencing of 16S ribosomal RNA was performed using the Illumina MiSeq platform.

### Sequencing and Data Analysis

A significant number of reads were generated by Illumina MiSeq high-throughput sequencer, which was called paired-end reads (raw data) and had a certain proportion of dirty data. The clean data and useful tags could be obtained through quality control and trimming subsequently. Then, the USEARCH method was used to cluster the tags into operational taxonomic units (OTUs) according to 97% similarity. The OTU species annotations were made using the SILVA database (https://www.arb-silva.de/) to obtain each sample’s community composition information.

According to the OTUs clustering results, on the one hand, species annotation was made for the representative sequence of each OTU to obtain the corresponding species information and species-based abundance distribution. Simultaneously, the OTUs were analyzed for abundance and alpha diversity calculation to get the species richness and evenness information in samples information between different groups. On the other hand, multiple sequence alignments could be performed on OTUs and constructed phylogenetic trees. The community structure differences of different samples and groups could be further obtained, displayed by principal coordinates analysis (PCoA) and other dimensionality reduction graphs and sample clustering trees. Finally, PICRUSt2 was used to predict the effect of community that we may better explore the function of intestinal bacteria and characterize better the potential role of the flora of different study populations. Statistics and visualization were obtained by Statistical Analysis of Metagenomic Profiles (STAMP).

### Statistics Analysis

All analyses were processed and plotted using QIIME version 1 (http://qiime.org/) and R (https://www.r-project.org/). *p* ≤ 0.05 was considered as the statistical significance and corrected using false discovery rate (FDR).

## Results

### Clinical Characteristics of T2DM and T2DM_GAN Patients

According to the WHO diagnostic criteria for T2DM, a total of 118 T2DM patients that cover 36 patients with T2DM_GAN and 19 normal people were recruited. All patients with T2DM_GAN were diagnosed with T2DM for more than 3 years, the longest even over 30 years. Finally, 73 patients with T2DM and 27 patients with T2DM_GAN completed the trial. Eighteen patients with diabetes did not meet the test due to incomplete clinical data including a lack of blood or stool samples.

The significantly different clinical data were calculated using the paired two-tailed Student’s t-test. Compared to the T2DM group, BMI, total bilirubin, unconjugated bilirubin, triglyceride, number of white and red blood cells, hemoglobin, and hematocrit were all strongly lower in the T2DM_GAN group (**Table 1**, *p* < 0.05). In addition, age and blood urea nitrogen were substantially higher.

### Illumina Sequencing Summary

To characterize the bacterial profiles present in these 119 subjects’ fecal microbiota, we performed next-generation sequencing of the V4 high-variable regions of the 16S rRNA gene with an Illumina MiSeq sequencing platform. The total number of paired-end reads obtained for 119 subjects were 10,535,570. After applying quality control and trimming, we received 8,637,657 high-quality sequences from diabetes patients (including T2DM and T2DM_GAN group) and 1,626,531 sequences from healthy controls, 97.42% of the total reads, and an average of 86,254 ± 7516 sequences per sample. Detailed information on the sequence results of each sample is presented in **Supplementary Table 1**.

### T2DM_GAN Significantly Altered the Gut Microbiota Diversity

Microbiota diversity analysis is valuable for quantifying the bacterial component and relative richness of a specific community. It includes alpha-diversity (intra-community), beta-diversity (inter-community), and gamma-diversity (total regional diversity) in the study of community ecology (Thukral et al., 2019). Alpha-diversity refers to species diversity within communities or within habitats, which can be measured by multiple indicators.

**Figure 1:**
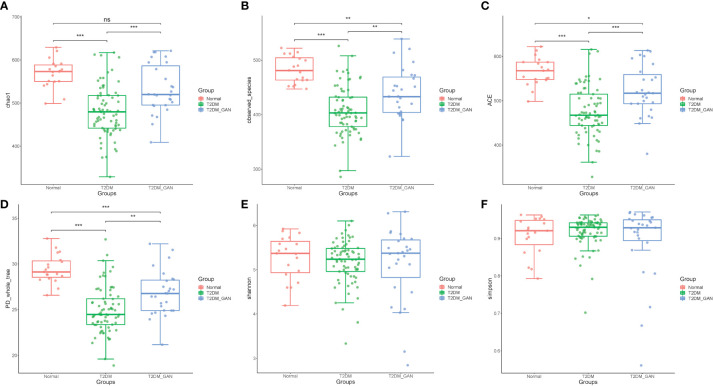
The alpha diversity of the gut microbiota among the subjects. **(A)** Chao1 index, **(B)** observed_species, **(C)** ACE index, **(D)** PD_whole_tree, **(E)** Shannon, and **(F)** Simpson. One-way ANOVA (normal *vs*. T2DM *vs*. T2DM_GAN group), multiple comparisons are performed using FDR correction: *p < 0.05, **p < 0.01, ***p < 0.001.

**Figure 2:**
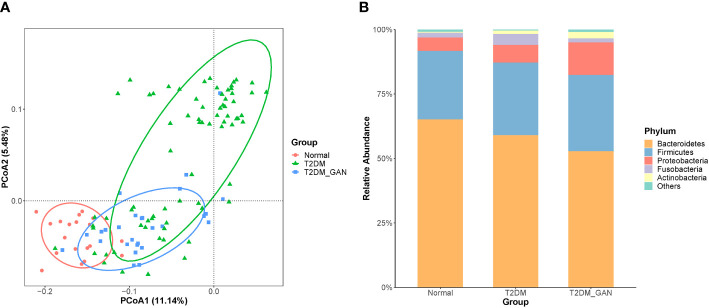
T2DM_GAN induce significant changes in the gut microbiota. **(A)** Unconstrained PCoA (for principal coordinates PCo1 and PCo2) with unweighted UniFrac distance shows that gut microbiota of 119 subjects formed three distinct clusters, which separate along the third coordinate axis (*p* = 0.001, mrpp). Ellipses cover 80% of the data for each group. **(B)** Distribution map of relative abundance of each group at the top 5 phyla.

In our study, the index of chao1, observed species, ACE, and PD_whole_tree were dramatically decreased in the T2DM and T2DM_GAN group compared to the normal group (**Figures 1A**–**D**). Furthermore, they were significantly increased in the T2DM_GAN group than in the T2DM group. No significant difference in Shannon and Simpson index were observed among the three groups (**Figures 1E, F**). In addition, a clear distinction was observed among the microbiota communities of the three groups, as shown in the PCoA plot (**Figure 2A**).

### T2DM_GAN Induced Significant Changes in the Gut Microbiota

To assess the impact of T2DM_GAN on gut microbiota composition, we analyzed the composition, abundance, and function of gut microbiota in fecal samples. Based on the clustering results, the vast majority (> 90%) of the sequences in all subjects of the study were found to belong to the three most abundant bacterial phyla, namely, Bacteroidetes, Firmicutes, and Proteobacteria (**Figure 2B**). In agreement with our results, previous investigations have also reported that those three phyla contribute to the majority of human gut microbiota (Larsen et al., 2010; De Filippo et al., 2010). Relevant differences were found in the proportions of three phyla: Bacteroidetes was more represented in normal than in T2DM and T2DM_GAN patients (65.19% versus 59.13% versus 52.56%, respectively), whereas *Firmicutes* and *Proteobacteria* were more abundant in T2DM and T2DM_GAN than in the normal group (26.61% versus 28.10% *versus* 29.59% and 5.2% *versus* 6.85% *versus* 12.61%, respectively). The remaining reads annotated to Fusobacteria, Actinobacteria, Tenericutes,Verrucomicrobia, and other taxonomic phyla (**Figure 2B**).

**Figure 3:**
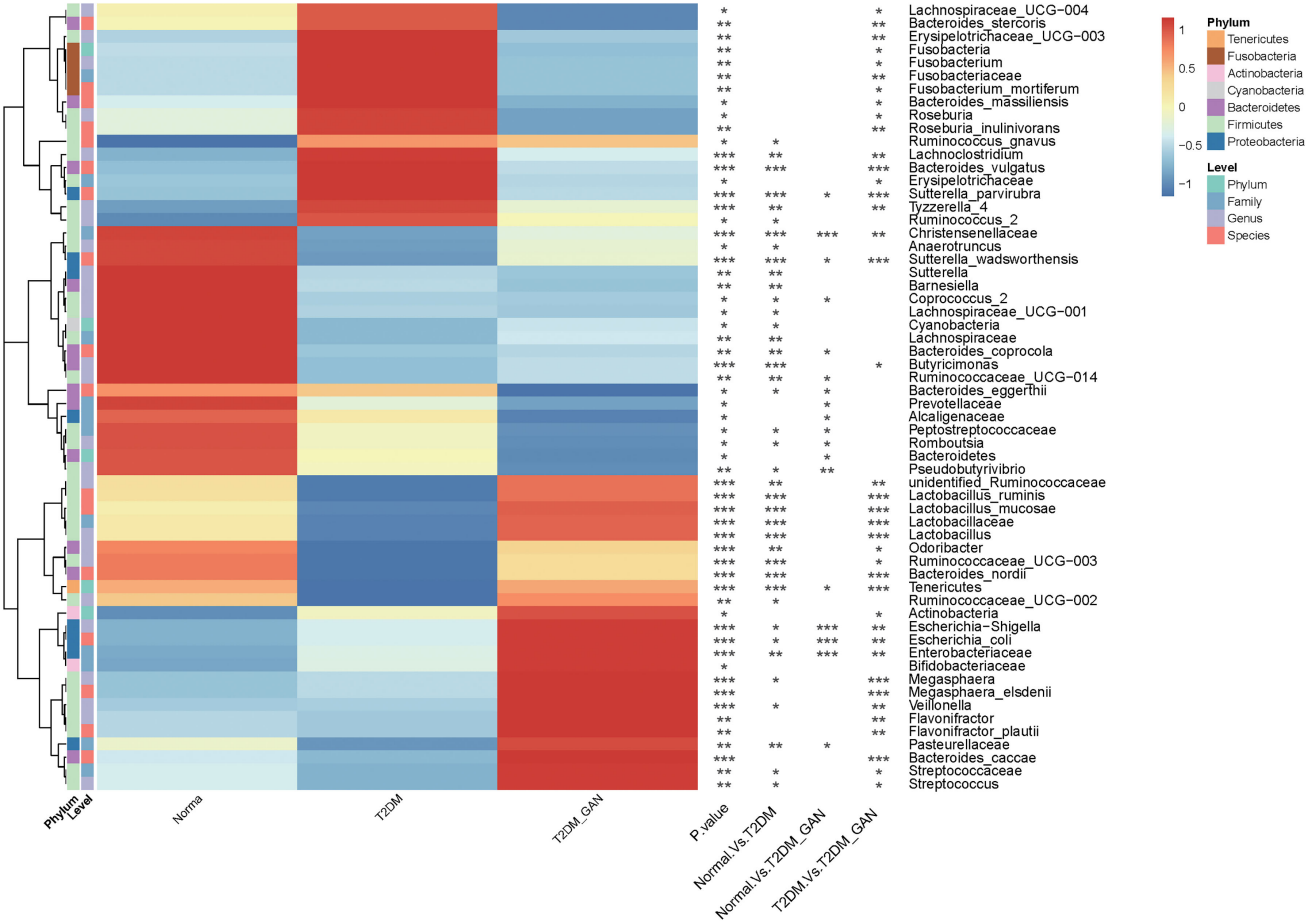
Gastrointestinal autonomic neuropathy exacerbates gut microbiota dysbiosis in adult patients with type 2 diabetes mellitus. Kruskal–Wallis test (normal *vs*. T2DM *vs*. T2DM_GAN group), multiple comparisons are performed using FDR correction: *p < 0.05, **p < 0.01, ***p < 0.001.

Kruskal–Wallis test was used to investigate the difference in gut microbiota composition among the three groups; multiple comparisons were performed using Wilcoxon signed-rank test and corrected using false discovery rate (FDR). A total of 60 marked different bacteria were identified at four levels, and their average relative abundance exceeded 0.1% (**Figure 3** and **Supplementary Table 2**). At phylum level, the proportion of Bacteroidetes was gradually decreased from normal to T2DM and then to T2DM_GAN, but the opposite trend was observed in Actinobacteria. The abundance of Fusobacteria was significantly higher in T2DM group than in T2DM_GAN group (**Supplementary Table 2**, *p* = 0.011), but the abundance of *Tenericutes* were significantly lower (**Supplementary Table 2**, *p* = 0.0009).

At the family level, 16 families were strongly different among the three groups. The abundance of Lactobacillaceae, Peptostreptococcaceae, and Alcaligenaceae were gradually decreased from normal to T2DM and then to T2DM_GAN of all. In addition, Lactobacillaceae, Enterobacteriaceae, Fusobacteriaceae, and Erysipelotrichaceae in the T2DM_GAN group were less abundant than that in the T2DM group, but the proportion of Christensenellaceae and Streptococcaceae were more abundant (**Supplementary Table 2**, *p* < 0.05).

At the genus level, there were 26 genera with significant difference among the three groups. Twenty-one genera in the normal group and 16 genera in the T2DM_GAN group were differentially abundant compared to the T2DM group, with 15 and 10 being upregulated, respectively (**Figure 3** and **Supplementary Table 2**). Most of the dramatically changed genera belonged to the phyla Firmicutes, Bacteroidetes, and Proteobacteria. Specifically, the order Clostridiales within the phylum Firmicutes accounted for 15 of the 26 different genera in the three groups (**Supplementary Table 2**).

At the species level, 30 species had an average relative abundance of more than 0.1%. Seventeen species were markedly different among the species of the three groups (**Figure 3** and **Supplementary Table 2**). In addition, 11 species in the normal group and 14 species in the T2DM_GAN group were differentially abundant as compared to the T2DM group (**Figure 3** and **Supplementary Table 2**), with 6 and 8 being upregulated, respectively. Interestingly, the species *Escherichia_coli* which belonged to Enterobacteriaceae family, was the top 3 species on all samples, and its abundance was much higher in the T2DM_GAN than in the other groups. Additional information for the strongly changed species has been listed in **Supplementary Table 2**.

**Figure 4:**
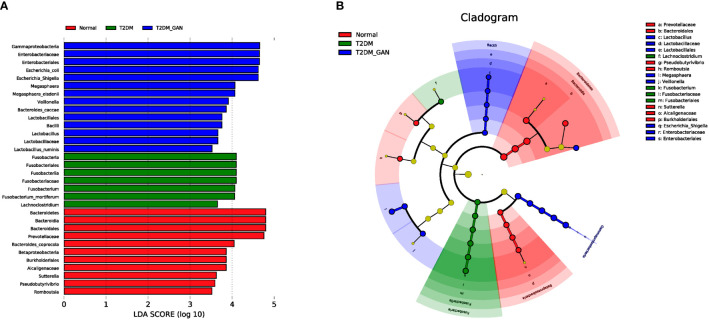
The biomarkers characteristic of the gut microbiota among the subjects. **(A)** Histogram of LDA scores: the height of the column represents the size of the LDA score, and the higher this value, the more significant the impact on the final classification (LDA > 3.5). **(B)** Cladogram showing differentially abundant taxonomic clades with an LDA score >3.5.

Biomarkers analysis using linear discriminant analysis effect size (LEfSe) indicated that T2DM patients were characterized by phylum Fusobacteria, class Fusobacteriia, order Fusobacteriales, family Fusobacteriaceae, genus *Fusobacterium*, genus *Lachnoclostridium*, and species *Fusobacterium_mortiferum* (**Figure 4**). In contrast, the class Gammaproteobacteria, order Enterobacteriales, family Enterobacteriaceae, genus *Escherichia-Shigella*, genus *Megasphaera*, species *E. coli*, species *Megasphaera_elsdenii*, and others were characteristic in the T2DM_GAN patients (**Figure 4**).

**Figure 5:**
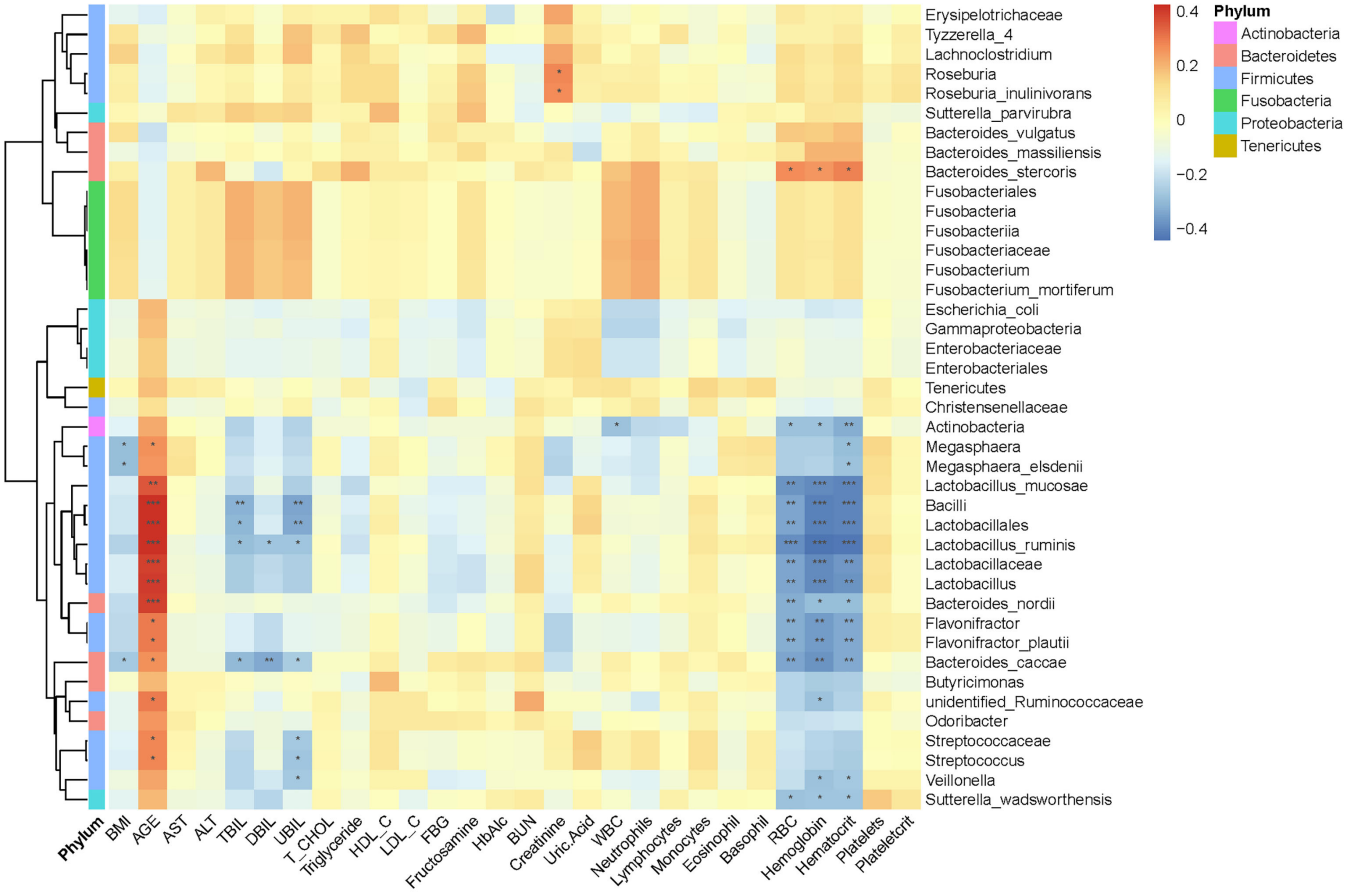
Association between clinical information and microbiota composition at the previously depicted differential bacteria. Notes: *p < 0.05, **p < 0.01, ***p < 0.001. BMI, body mass index; ALT, alanine aminotransferase; AST, aspartate aminotransferase; TBIL, total bilirubin; DBIL, direct bilirubin; UBIL, unconjugated bilirubin; T_CHOL, total cholesterol; HDL_C, high-density lipoprotein cholesterol; LDL_C, low-density lipoprotein cholesterol; FBG, fasting blood glucose; HbA1c, glycosylated hemoglobin; BUN, blood urea nitrogen; WBC, white blood cells; RBC, red blood cells.

The above analysis have shown that there were differences in gut microbial diversity, complexity, and composition between the patients with T2DM and T2DM_GAN. Whereafter, we identified a few diverse species and taxonomic communities that may play a dominant role in their respective populations. To find the taxonomic bacteria, we performed the Spearman correlation between bacteria and clinical data and displayed as heat map that could examine the relationship between clinical factors and species in T2DM and T2DM_GAN patients (**Figure 5**). The results showed that Actinobacteria, Bacilli, Lactobacillales, Lactobacillaceae, *Lactobacillus*, *Lactobacillus_ruminis*, *Lactobacillus_mucosae*, *Flavonifractor*, *F. plautii*, *Bacteroides_nordii*, and *Bacteroides_caccae* were significantly negatively correlated with several clinical data including red blood cells, hemoglobin, and hematocrit, and opposite with age (**Figure 5**). It is worth noting that most of these bacteria belong to class Bacilli.

### Effect of T2DM_GAN on Metabolic Pathways

The functional metagenomic analysis inferred using PICRUSt2 (Douglas et al., 2020) (phylogenetic investigation of communities by reconstruction of unobserved states 2) examined how the bacterial functional profiles differed among disease groups. As we know, PICRUSt2 is a software tool that predicts the functional profile of a microbial community based on 16S rRNA sequences (Douglas et al., 2020). It provides a starting point for understanding functions potentially represented within a microbial community.

**Figure 6:**
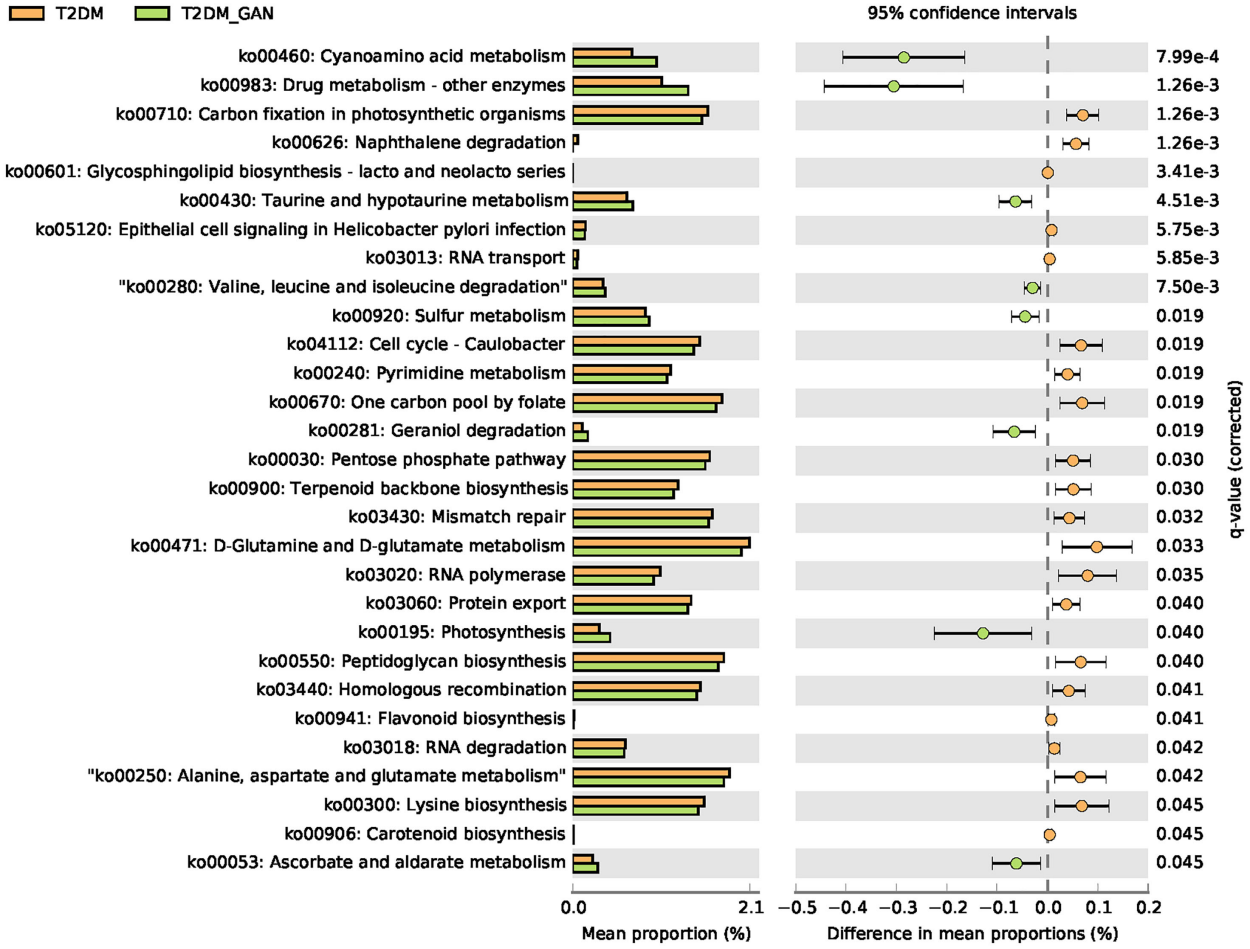
The significantly and differentially pathway between T2DM and T2DM_GAN patients. The left X-axis represents different groups, the Y-axis represents the average relative abundance of a species in different groups, and the right represents the confidence interval and *p*-value.

**Figure 7:**
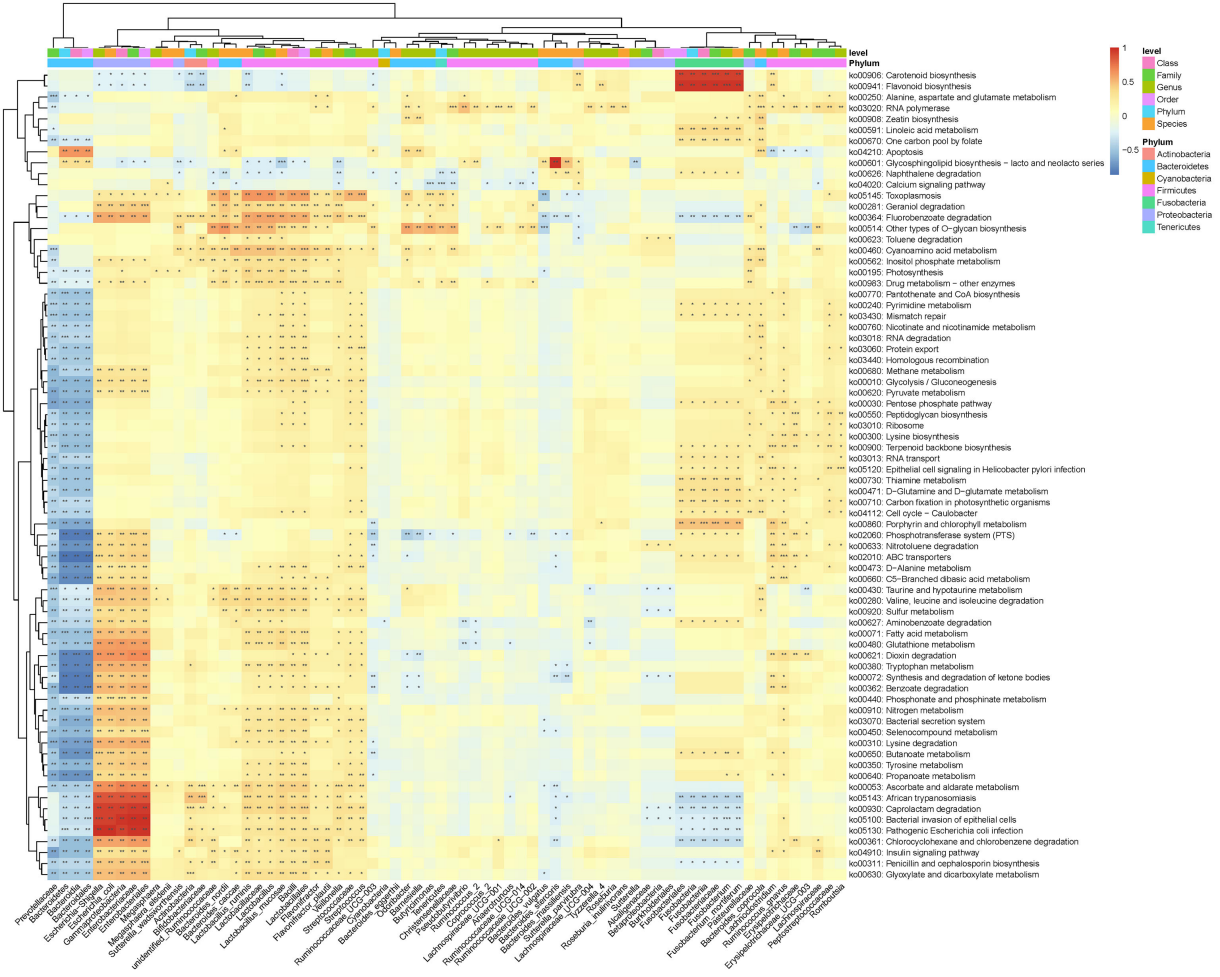
Association between differential KEGG pathways and microbiota composition at the previously depicted differential bacteria. Notes: *p < 0.05, **p < 0.01, ***p < 0.001.

In this study, PICRUSt2 predicted a total of 7,060 Kyoto Encyclopedia of Genes and Genomes (KEGG) ortholog (KO) genes from the entire data set and annotated 170 KEGG pathways. These pathways were mainly distributed in the metabolism (81.11%), Genetic Information Processing (12.97%), Cellular Processes (3.15%), Environmental Information Processing (2.02%), Organismal Systems (0.46%), and Human Diseases (0.30%) of the KEGG pathway at level 1 (**Supplementary Figure 1**). In addition, we used STAMP (Parks et al., 2014) software to depict the general metabolic pathways and compare microbiota functions among the three groups of subjects, highlighting the significant difference in the distribution of metabolic pathways (**Figure 6**; **Supplementary Figures 2** and **3**). In T2DM and T2DM_GAN patients, the predicted KEGG pathways that were significantly differently related to two metabolic pathways involved in amino acid metabolism (“alanine, aspartate and glutamate metabolism,” “valine, leucine and isoleucine degradation,” “lysine biosynthesis,” “taurine and hypotaurine metabolism,” “D-glutamine and D-glutamate metabolism”) and carbohydrate metabolism (pentose phosphate pathway, peptidoglycan biosynthesis, glycosphingolipid biosynthesis—lacto and neolacto series) (**Figure 6**). Overall, the microbial communities presented in the two groups could be distinguished based on their functions. A further discovery was that epithelial cell signaling in *Helicobacter pylori* infection was significantly reduced in T2DM_GAN patients (**Figure 6**). Additionally, gut microbiota biomarkers of T2DM patients may be involved in both carotenoid and flavonoid biosyntheses. The gut microbiota biomarkers of T2DM_GAN patients may be associated with both bacterial invasion of epithelial cells and pathogenic *E. coli* infection (**Figure 7**).

## Discussion

The composition of the gut microbiota is thought to change during the development of diabetes. However, few reports have utilized sequencing techniques to investigate the dynamical changes in gut microbiota composition of patients with T2DM_GAN. In this study, we demonstrated that GAN exacerbates gut microbiota dysbiosis in adult patients with T2DM and suggested that the development of diabetes can be affected by the gut microbiota. The present study indicated that the intestinal flora could distinguish the normal group from the diabetes patients, including patients with T2DM and T2DM_GAN. Furthermore, our results showed that the diversity of diabetic patients is lower than that of the normal individuals. This finding was consistent with previous studies; low richness of gut microbiota had been reported in patients with inflammatory bowel disorder (Qin et al., 2010), elderly patients with inflammation (Claesson et al., 2012), and obese individuals (Le Chatelier et al., 2013), and increased risk of pre-diabetes, T2DM, and ischemic cardiovascular disorders (Le Chatelier et al., 2013).

Microbiological testing of stool samples indicated that there are qualitative and quantitative differences in the composition of the gut microbiota among the subjects. Despite considerable variation found in different individual’s microbiota, the predominant phyla of bacteria in subjects were Bacteroidetes and Firmicutes, which account for >90% of the total gut microbiota in normal people. The rest of the phyla were Proteobacteria, Fusobacteria, Actinobacteria (e.g., Gram-positive anaerobic bacilli *Bifidobacterium*), and Verrucomicrobia (e.g., Gram-negative anaerobic oval-shaped bacteria *Akkermansia*) (Salamon et al., 2018). These results corroborated the findings of a great deal of the previous work (Shen et al., 2013; Blandino et al., 2016; Salamon et al., 2018).

The dominant bacterial species largely determine the function of the gut microbiota community. Thus, understanding the species composition at different taxonomic levels can effectively interpret the formation and change of the community structure in T2DM_GAN patients. In the present study, we found that the most significant difference in intestinal microbiota between T2DM and T2DM_GAN patients mainly came from the phyla Fusobacteria and Proteobacteria (**Figure 2B** and **Supplementary Table S2**).

On the one hand, we first discussed the effect of Proteobacteria in detail. The T2DM_GAN patients were represented by enriched class Gammaproteobacteria, order Enterobacteriales, family Enterobacteriaceae, genus *Escherichia-Shigella*, genus *Megasphaera*, species *E. coli*, species *M. elsdenii*, and others (**Figure 4**). It is generally known that Proteobacteria blooms in the intestine, reflecting an unstable microbial community structure and a host disease state. The members of Proteobacteria, a rich source of lipopolysaccharides (LPS) (Round and Mazmanian, 2009), lead to diabetes through playing a vital role in increasing the level of proinflammatory cytokines and impairing pancreatic beta-cell function (Han et al., 2018). Several reports have shown increased Proteobacteria and its members associated with inflammatory bowel disease (IBD), irritable bowel syndrome (IBS), and ileal Crohn’s disease (CD) (Morgan et al., 2012; Gevers et al., 2014; Lane et al., 2017; Salem et al., 2018). For example, there was an increase in Enterobacteriaceae, Pasteurellacaea, Veillonellaceae, and Fusobacteriaceae in pediatric CD patients (Gevers et al., 2014). Moreover, there was an increased abundance of pathogenic anaerobic organisms including Enterobacteriaceae, Fusobacteriaceae, *Escherichia_coli*, and *Fusobacterium* in IBS patients (Gevers et al., 2014; Lane et al., 2017). Meanwhile, Salem et al. (2018) summarized the evident differences in gut microbiota between IBS patients and normal volunteers in that Enterobacteriaceae, Proteobacteria, Veillonella, and Firmicutes aerobes increased and aerobic bacteria decreased. In conclusion, species belonging to the phyla Proteobacteria would be associated with gastrointestinal symptoms. It is interesting to note that the correlation of this study also found that the dominant bacteria in T2DM_GAN patients, Proteobacteria phylum and its members, were likely to be involved in bacterial invasion of epithelial cells and pathogenic *E. coli* infection.

On the other hand, we will gain insight into Fusobacteria and the related effects. The phylum Fusobacteria, class Fusobacteriia, order Fusobacteriales, family Fusobacteriaceae, genus *Fusobacterium*, genus *Lachnoclostridium*, and species *F. mortiferum* were characteristics in the T2DM patients (**Figure 4**). Moreover, prior studies have noted the importance of *Fusobacteria* phylum and its members in other diseases. For instance, the research of Ahmad et al. also detected an abundance of phyla Fusobacteria in the obese-T2DM samples (Ahmad et al., 2019). Tahara et al. have revealed that *Fusobacterium* is a clinicopathological feature for UC patients in Japan (Tahara et al., 2015). The invasive ability of Fusobacteria was positively correlated with IBD severity of the host (Weingarden and Vaughn, 2017; Ma et al., 2018). Studies have shown that the class Fusobacteria is a kind of adherent and invasive bacteria (Yang et al., 2020) and plays a crucial role in energy production, mounting adhesiveness to host epithelial cells, and inflammation responses. Its high abundance was contributing significantly to T2DM and other diseases (Ahmad et al., 2019). Further analysis found that those microbiota, represented by phylum Fusobacteria in T2DM patients, may be involved in carotenoid biosynthesis and flavonoid biosynthesis. Carotenoids are one of the lipids that are bioactive and well recognized for their antioxidant activities and regulation of cellular growth and immune response (Al-Ishaq et al., 2020). Flavonoids, natural phenolic compounds found abundantly in fruits and vegetables, were reported to have potentially anti-neurodegenerative, anti-inflammatory, anti-cardiovascular activities and anticancer abilities (Al-Ishaq et al., 2021). In addition, several studies have shown that *Fusobacterium_nucleatum* is an emerging pathogen, which has been implicated as a causal microbe in several diseases of the gastrointestinal tract, including CD and colorectal cancer (Cochrane et al., 2020). Therefore, according to these data, we boldly inferred that Fusobacteria and its members might influence occurrence and progression of GAN in T2DM patients. Although we know that this microbe can cause illness, how it does this remains enigmatic. Further investigations are needed to identify the detailed mechanism.

Subsequently, we found that pathways mainly related to amino acid metabolism and carbohydrate metabolism were significantly different between the T2DM and T2M_GAN patients. Several previous studies have shown that essential amino acids metabolic pathways seem to be associated with obesity and insulin resistance (Ridaura et al., 2013; Sanmiguel et al., 2015). In addition, because the intestine is the main site for nutrient absorption, when the bacteria in the intestine are significantly increased, they often cause gastrointestinal symptoms, affect the absorption of carbohydrates and fats, and worsen intestinal inflammation (Yao et al., 2016). In reviewing the literature, amino acids could affect immune responses either directly or indirectly through their metabolites (Li et al., 2007). Therefore, changes in these amino acid metabolism pathways could be a result of the body’s antiviral response. Although the microbiota functional differences among the normal, T2DM, and T2DM_GAN groups were not experimentally confirmed in our study, the predicted metagenomic pathways had significant differences, which could provide meaningful information for the microbial role of relating gastrointestinal symptoms.

Notably, although this study was the first to compare the gut microbiota between the patients with T2DM and T2DM_GAN, it still has certain limitations. First, the sample distribution of the subjects was uneven, and there were big differences in age and BMI among patients. However, this difference was mainly due to the clinicopathological characteristics of T2DM and T2DM_GAN patients. T2DM_GAN is an intestinal complication of T2DM, and the long-term disease state is one of its causes. In addition, patients with T2DM_GAN may experience a variety of burdensome symptoms, including dysphagia, indigestion, pain, abdominal distension, diarrhea, constipation, fecal incontinence, vomiting, and weight loss, all of which can adversely affect the quality of life. Studies have reported that 70 years old was considered the threshold age for defining an individual as elderly because the gut microbiota may undergo major changes at this time (Odamaki et al., 2016). Second, another limitation was a lack of clinical data for the normal people in the study. Unfortunately, the blood samples of normal individuals could not be collected during the study period. Therefore, we only performed a correlation analysis for fecal samples and clinical data between T2DM and T2DM_GAN. The microbial community’s composition depends on its colonization location and is affected by other several factors, such as the genetic, living environment (continent, climate), age, and diet (Vallianou et al., 2018). A limitation of our study is the lack of detailed nutritional data for participants. However, based on the same hospital participators, we could assume that their nutrition was no different. Therefore, to further determine the sequential changes in gut microbiota in T2DM_GAN patients, larger, more comprehensive, and complete experiments are needed.

Despite these limitations, our data still provided a novel better understanding of gut microbiota’s role in developing T2DM and its complication. The gut microbiota may be related to the pathological state or severity of diabetes, and microflora interventions; for example, probiotics, prebiotic treatments, and stool transplantation was of great significance for treating and preventing complications of T2DM. Especially in the stage of T2DM, targeting related bacteria may better prevent the occurrence and development of complications.

## Conclusions

Gastrointestinal autonomic neuropathy exacerbates gut microbiota dysbiosis in adult patients with T2DM, and the most significant difference between them mainly came from the phyla Fusobacteria and Proteobacteria. The former is a kind of Gram-negative bacteria, which was the most abundant in the T2DM population. The latter is an LPS-producing bacteria phylum, which was dramatically more in the T2DM_GAN group than in other groups. To be specific, T2DM patients were characterized by phylum Fusobacteria, class Fusobacteriia, order Fusobacteriales, family Fusobacteriaceae, genus *Fusobacterium*, genus *Lachnoclostridium*, and species *F. mortiferum*. In contrast, the class Gammaproteobacteria, order Enterobacteriales, family Enterobacteriaceae, genus *Escherichia-Shigella*, genus *Megasphaera*, species *E. coli*, species *M. elsdenii*, and others were characteristic in the T2DM_GAN patients (**Figure 3**). Furthermore, there were significant changes in pathways mainly related to amino acid metabolism and carbohydrate metabolism in T2DM and T2M_GAN patients. A characteristic of gut microbiota in T2DM patients is that they may be involved in carotenoid and flavonoid biosynthesis and that of T2DM_GAN patients may be involved in bacterial invasion of epithelial cells and pathogenic *E. coli* infection.

## Data Availability Statement

The data presented in the study are deposited in the github: https://github.com/duyuhui/Gut-microbiota-of-T2DM_GAN

## Ethics Statement

XK, HS, and YK conceived the research project; YD, QN, YL, YK, LG, XH, MC, FY, JH, SZ, JZ and FY performed the experiments; YD analyzed the data, and wrote the draft; YK revised the manuscript; XK, HS, and YK supervised the work; All authors read and approved the final manuscript.

## Author Contributions

XK, HS, and YK conceived the research project; YD, QN, YL, YK, LG, XH, MC, FY, JH, SZ, JZ and FY performed the experiments; YD analyzed the data, and wrote the draft; YK revised the manuscript; XK, HS, and YK supervised the work; All authors read and approved the final manuscript.

## Conflict of Interest

The authors declare that the research was conducted in the absence of any commercial or financial relationships that could be construed as a potential conflict of interest.

## Publisher’s Note

All claims expressed in this article are solely those of the authors and do not necessarily represent those of their affiliated organizations, or those of the publisher, the editors and the reviewers. Any product that may be evaluated in this article, or claim that may be made by its manufacturer, is not guaranteed or endorsed by the publisher.

## Supplementary Material

Supplementary Figure 1 Pie chart shows the distribution of KEGG metabolic pathways of level 1.

Supplementary Figure 2 The significantly and differentially pathway between Normal people and T2DM patients. The left X-axis represents different groups, the Y-axis represents the average relative abundance of a species in different groups, and the right represents the confidence interval and p-value.

Supplementary Figure 3 The significantly and differentially pathway between Normal people and T2DM_GAN patients. The left X-axis represents different groups, the Y-axis represents the average relative abundance of a species in different groups, and the right represents the confidence interval and p-value.

Supplementary Table 1 Illumina sequencing summary.

Supplementary Table 2 GAN exacerbates gut microbiota dysbiosis in adult patients with T2DM. Notes: Kruskal-Wallis Test is used to compare the three groups (Normal vs T2DM vs T2DM_GAN group, kruskal pvalue). And multiple comparisons are performed using Wilcoxon Signed Rank Test and corrected using FDR.
