# Supplementary figures and images for: Gastrointestinal Autonomic Neuropathy Exacerbates Gut Microbiota Dysbiosis in Adult Patients With Type 2 Diabetes Mellitus
Source: Front Cell Infect Microbiol. 2022 Feb 8;11:804733. doi: 10.3389/fcimb.2021.804733 (PMC8861497; doi:10.3389/fcimb.2021.804733)

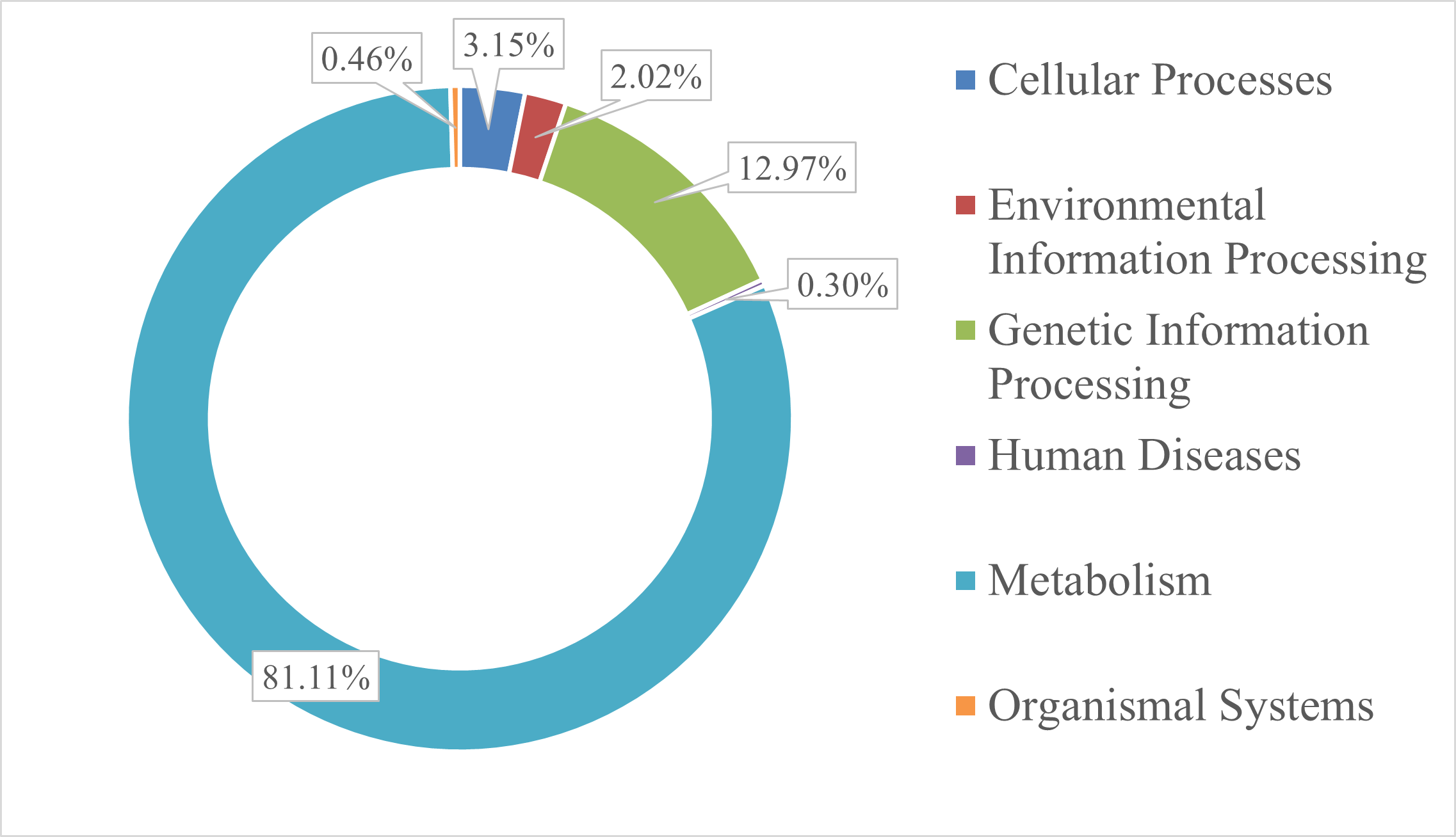

Supplement: Supplementary Figure 1 — Pie chart shows the distribution of KEGG metabolic pathways of level 1. [file Image_1.png]

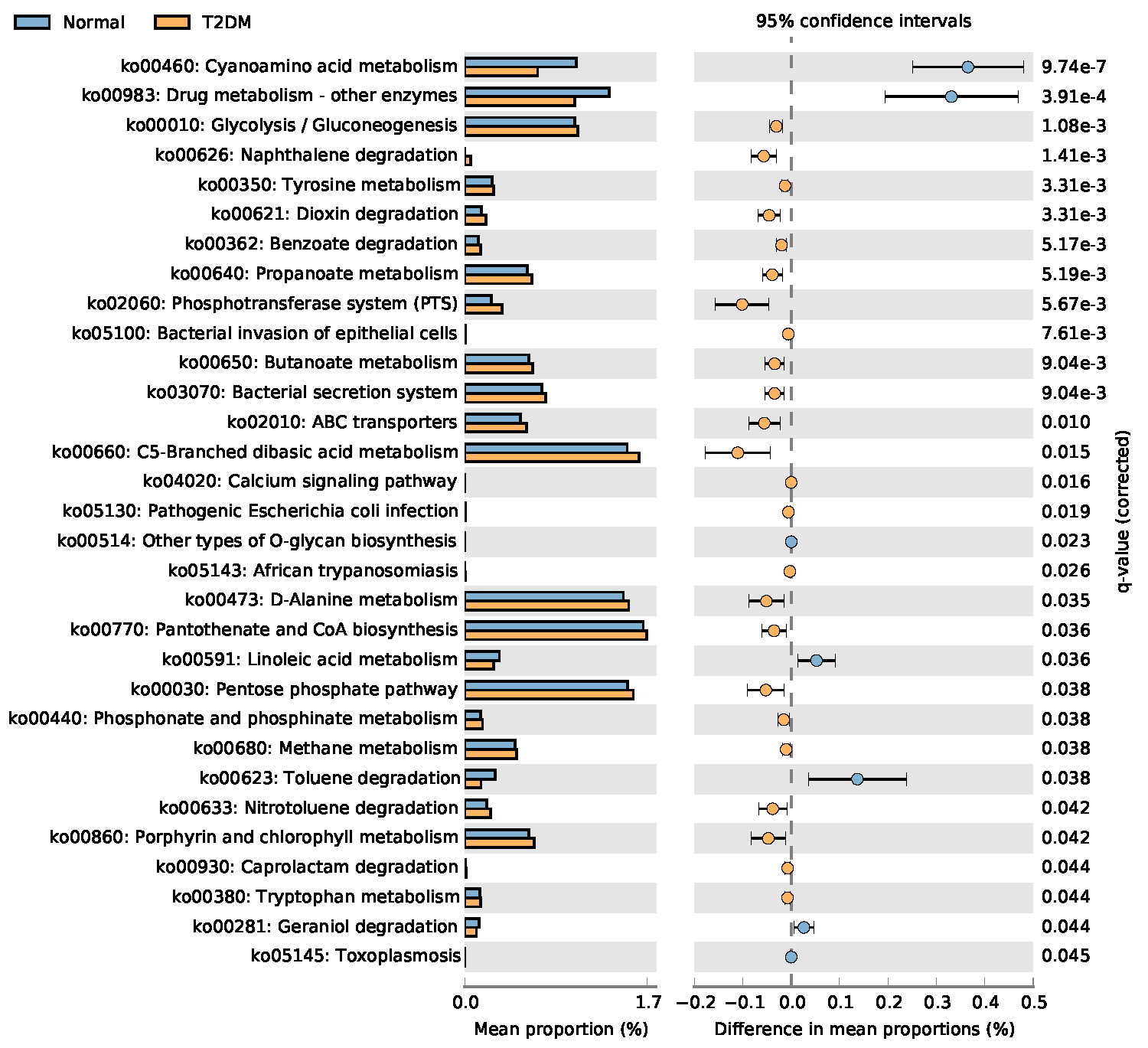

Supplement: Supplementary Figure 2 — The significantly and differentially pathway between Normal people and T2DM patients. The left X-axis represents different groups, the Y-axis represents the average relative abundance of a species in different groups, and the right represents the confidence interval and p-value. [file Image_2.tiff]

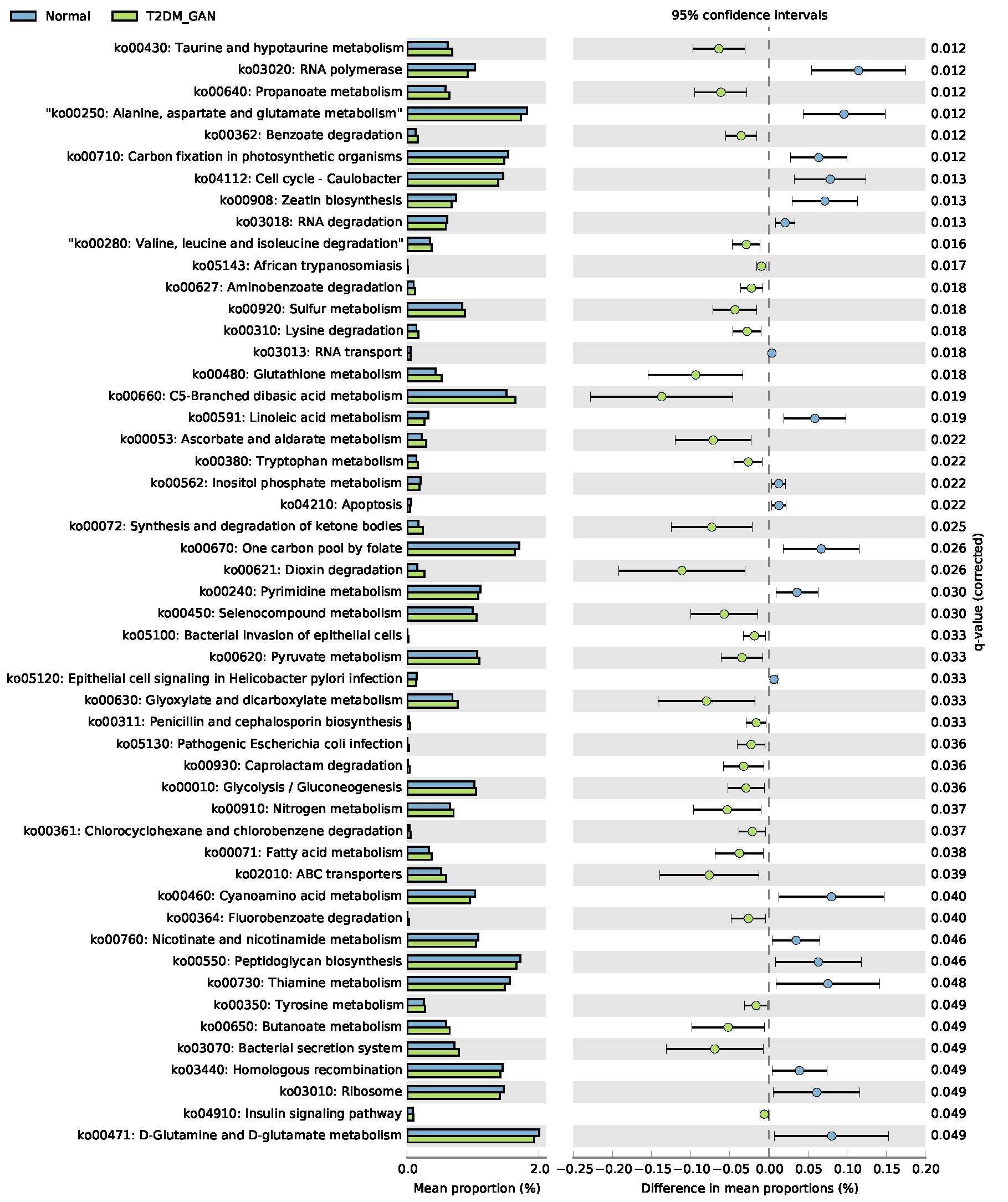

Supplement: Supplementary Figure 3 — The significantly and differentially pathway between Normal people and T2DM_GAN patients. The left X-axis represents different groups, the Y-axis represents the average relative abundance of a species in different groups, and the right represents the confidence interval and p-value. [file Image_3.tiff]
